# Too humble for words

**DOI:** 10.1007/s11098-023-02031-4

**Published:** 2023-09-11

**Authors:** Neil Levy

**Affiliations:** 1 Department of Philosophy, Macquarie University, Sydney, NSW 2109 Australia; 2 Uehiro Centre for Practical Ethics, University of Oxford, Oxford, OX1 1PT UK

**Keywords:** Epistemic humility, Virtue epistemology, Illusion of explanatory depth, Individualism

## Abstract

It’s widely held that a lack of intellectual humility is part of the reason why flagrantly unjustified beliefs proliferate. In this paper, I argue that an excess of humility also plays a role in allowing for the spread of misinformation. Citing experimental evidence, I show that inducing intellectual humility causes people inappropriately to lower their confidence in beliefs that are actually justified for them. In these cases, they manifest epistemic humility in ways that make them epistemically worse off. I argue that epistemic humility may fail to promote better beliefs because it functions for us against the background of our individualistic theory of responsible epistemic agency: until we reject such theories, intellectual humility is as much a problem as a solution to epistemic ills. Virtue epistemology is inadequate as a response to unjustified beliefs if it does not look beyond the virtues to our background beliefs.

There’s too much humility around. In particular, there’s too much *intellectual* humility (IH) around, or so I’m going to argue. In arguing that there’s too much IH around, I am swimming against strong currents: it’s widely held that a *lack* of IH is responsible for a range of epistemic ills. The apparent rise in and even respectability of bizarre beliefs, like wild conspiracy theories, and of beliefs at odds with the scientific consensus has many causes, but many people think that a lack of IH is one part of the problem. Persisting in false beliefs in the face of strong evidence against them isn’t remotely humble: it’s arrogant. And arrogance is the contrary of humility (or, in more Aristotelian terms, one of the two vices that flank the virtue).^1^

It's very plausible that there *is* too much intellectual arrogance around; whether or not that’s true, there’s also too much IH. The induction of IH leads ordinary people too quickly to abandon their beliefs in the face of contrary evidence, and this disposition probably plays a role in explaining many of the epistemic ills mentioned above. The cause, I will argue, is genuinely IH, and not some other disposition that might be mistaken for it; IH is therefore epistemically harmful.

The conclusion that it’s IH (and not, say, epistemic servility) that is epistemically harmful is highly counterintuitive. I’ll moderate the counterintuitiveness of the conclusion by identifying a different *ultimate* culprit: epistemic individualism. It is only because people are typically epistemic individualists that the induction of IH is epistemically harmful. Inducing IH brings people to act more consistently with their individualistic commitments, but in so doing they lose knowledge. Whatever role a deficit in IH might play in our current epistemic predicament, increasing it threatens to worsen matters. Instead, we need to target our background commitment to epistemic individualism.

## The nature of intellectual humility

There’s an immediate problem with arguing that there’s too much IH around: there’s nothing like a consensus in the literature on what IH consists in.^2^ On some views, IH is metacognitive: the intellectually humble person is the person who calibrates their attitudes toward their beliefs in a way that reflects the evidence (Church, 2016; Hazlett, 2012). Other views are concerned with our attitude toward ourselves or some aspect of ourselves. Roberts and Woods (2007) take IH to consist in an unusually low level of concern for one’s status vis-à-vis other agents (see Tanesini, 2021a, 2021b, for a related view). Driver (2001) takes it to consist in the underestimation of one’s abilities.^3^ Perhaps most influentially, Whitcomb et al. (2017) have argued that IH consists in *owning* our intellectual limitations, where this consists in being mindful of and accepting these limitations and accepting, too, that their negative outcomes are due to these limitations. On all these views, IH is inward-looking: it concerns how the agent regulates her intellectual life. On other views, it is essentially outward-looking, consisting in concern for the intellectual flourishing of others and respect for them as epistemic agents (Priest, 2017). There are also hybrid views; for instance Tanesini’s complex account, on which IH consists in both modesty and acceptance of one’s limitations, which she sees as distinct psychological characteristics that tend to co-occur because possession of one without the other gives rise to cognitive dissonance (Tanesini, 2018).

The broad variety of views I have just surveyed is by no means exhaustive. I won’t attempt to adjudicate between them, or others I have not mentioned. I don’t think that’s necessary for my purposes. While different accounts give rise to different verdicts in some cases, it’s a condition of adequacy that they give the right result in clear cases; accordingly, they overlap considerably on the kinds of cases they assess as exemplifying or manifesting IH, on the one hand, and its absence, on the other. They also overlap considerably on the dispositions that are characteristic of the epistemically humble person: dispositions the lack of which would cast doubt on the claim that they possessed the virtue.^4^ By focusing on such cases and dispositions, I can avoid settling on the right account of IH. The kinds of cases I will present (drawn not from thought experiments, but from the laboratory) seem at least prima facie to involve the loss of justified belief or even knowledge arising from a disposition toward IH.

It's a truism that intellectually humble people moderate their views in the light of evidence. In particular, they reduce their confidence in a proposition upon learning that there is a defeater for that proposition (unless they also learn that that defeater is itself defeated). This is a truism that all sides are quick to acknowledge.^5^ For Whitcomb and colleagues, for example, intellectual humility “increases a person’s propensity to revise a cherished belief or reduce confidence in it, when she learns of defeaters” (Whitcomb et al., 2017: 524). Defeaters feature centrally among what Kidd (2016) calls *confidence constraints*, and as he notes, ignoring such constraints is “partly definitive of the vices associated with a lack of humility” (397). In particular, the intellectually humble person will moderate her confidence if she’s unable to offer grounds for her assertions (Kidd, 2016: 319). In the face of evidence that she does not understand its grounds, the truly humble person “admits to her lack of understanding and either seeks assistance or thinks harder about the topic” (Tanesini, 2018: 405).

The cases I will present provide evidence that ordinary people do indeed reduce their confidence in their beliefs when they realize that their grasp of its grounds is weaker than they had thought. That might seem like good news. I’m going to suggest that it’s not the good news we might take it to be. If the evidence generalizes, as we should think it does, it shows that we tend to be conciliatory when we shouldn’t be; such a disposition to conciliation may play a role in explaining our epistemic predicament.

## Humbling beliefs

We’re highly susceptible to an *illusion of explanatory depth* (Mills & Keil, 2004; Rozenblit & Keil, 2002): when something is familiar to us, we often take ourselves to understand how it works at a mechanistic level much better than we actually do. The relevant experiments usually proceed as follows. First, participants are asked to rate their level of understanding of some familiar device or mechanism (how a zipper works; how the flush mechanism on a toilet works; how a piano produces sound, and so on). They are then asked to describe in detail exactly how it works, step by step. Finally, they’re asked to rate their level of understanding of the device again. The consistent finding is that participants discover that their mechanistic understanding of the device is much vaguer than they’d thought; consequently, their second-stage rating of their understanding is significantly lower than at the first stage.

Sloman and colleagues hypothesized that revealing the illusion of explanatory depth in the political domain to experimental participants might serve to reduce their degree of extremism (Fernbach et al., 2013; Sloman & Vives, 2022). They asked their participants to report their attitudes toward policy questions (e.g., *should student debt be cancelled*; *should the cost of prescription medicines be capped*; *should there be stricter background checks for those seeking to purchase a firearm*; *should the qualifying conditions for US citizenship be more demanding*). Participants were also asked to judge their level of understanding of the policies. They were then (in the condition of interest) asked to provide a mechanistic explanation for just *how* the policies would achieve their goals, before having their attitudes toward the policies measured again.

The experimenters found that the requirement to provide explanations reduced participants’ assessment of their own understanding of the policy as expected. Moreover, it also reduced extremism: that is, it shifted people from the extreme ends of the scale (“strongly agree;” “strongly disagree”) to more moderate positions.

While this finding has been replicated multiple times (e.g., Johnson et al., 2016; Vitriol & Marsh, 2018; Voelkel et al., 2018), Crawford and Ruscio (2021) failed to replicate the key finding using the same policies and procedures that Fernbach et al. (2013) had used. They found that asking people to explain how the policies they supported would achieve their goals indeed reduced their reported level of understanding, but not their level of support for them. Sloman and Vives (2022) is aimed, in part, at explaining this failure to replicate, and indeed why a reduction in extremism as a consequence of the manipulation should not be expected to generalize to all policy issues.

Sloman and Vives argue that even when a policy is explicitly aimed at some end, support for that policy only sometimes depends on our sense of its consequences. Some policies are supported on different grounds: because support for them is seen as definitive of being an authentic member of a particular party or faction, or because the person identifies with it, for example. Policies often move from the first category to the second: those who identify with one side of a policy debate might become wedded to it, regardless of whether it achieves the goals for which it was initially adopted. Suppose it was definitively shown that making abortion illegal significantly increased the number of abortions performed. We wouldn’t expect Republican support for a ban to drop, at least not anytime soon. Similarly, if it was shown definitively that affirmative action policies decreased the number of people belonging to historically underrepresented groups hired to important positions, Democrats would be slow to abandon them. Because this is the case, Sloman and Vives argue, we shouldn’t expect the manipulation they used to be effective in reducing support for all policies aimed at a goal. It is only if the policy is aimed at a goal *and* support has not yet become entrenched that the manipulation will be effective.

Sloman and Vives (2022) aim to test this account, using four different policies. “*Should student debt be cancelled*” and “*should the cost of prescription medicines be capped*” are policy questions attitudes to which would genuinely be based on perceptions of their perceived consequences, they argue.^6^ But “*should there be stricter background checks for those seeking to purchase a firearm*” and “*should the qualifying conditions for US citizenship be more demanding*” are questions that are assessed in terms of their expression of what Sloman and Vives call *protected values*, where a protected value cannot be traded off for other goods and is non-negotiable. As expected, the manipulation was successful in bringing people to realize that they understood the mechanisms involved in all four policies much less well than they had previously thought, but it reduced extremism only for “student debt” and “capping medicines”. Why, then, did Crawford and Ruscio (2021) fail to decrease support for the very same policies selected by Fernbach et al. (2013)? Sloman and Vives point to the 8 years that separate the two studies. Given the tendency of partisan policies to become entrenched over time, we should expect policies that were once supported on consequentialist grounds to become resistant to the manipulation.

I’m sympathetic to Sloman and Vives’ argument. However, it’s enough for my purposes if the experimental manipulation often reduces support for policies, whatever the causal mechanism(!) Given the preponderance of evidence, right now we should think that it does. I turn, then, to assessing the epistemic significance of the finding that exposing the illusion of explanatory depth reduces confidence in policies. Should we see the reduction in confidence that follows a demonstration of the shallowness of their grasp of the mechanisms in play as an improvement in the overall epistemic position of the participants?

Fernbach et al. (2013) and Sloman and Vives (2022) present their findings as good news. They take themselves to have shown that it is possible to use the manipulation as a “debiasing procedure” (Fernbach et al., 2013: 945). It works, they themselves argue, by inducing “a kind of intellectual humility” in participants (Sloman & Vives, 2022: 1). We might therefore interpret their results as suggesting that IH is a virtue that we might call upon to address at least some aspects of what I above called our epistemic predicament.

But we shouldn’t be too quick to celebrate IH. Sloman and colleagues argue, in effect, that inducing IH has salutary effects because it reveals to their participants that their grounds for their attitudes were inadequate: they didn’t really understand how these policies are supposed to bring about their goals. But participants shouldn’t—and don’t—base their attitudes toward policies on their own assessment of how they work. Our attitudes to policies, even to those that are unambiguously consequentialist in character (that is, justified on the grounds that they are the best means to bring about some desired end, like policies aimed at reducing carbon emissions), are rarely warranted on the basis of our grasp of the causal mechanisms involved. Consider support (or, for that matter, opposition) to policies that mandate or encourage vaccination against Covid, or measles. *Very* few of us have the expertise in medicine to grasp the causal mechanisms involved in vaccines sufficiently well for that understanding to ground our assessment of the relative benefits of vaccination. Worse, such knowledge is insufficient to justify support for the policy: in addition to being able to assess the efficacy of vaccines, we’d also need to know a great deal concerning how they are produced, and shipped, and delivered, and about the opportunity costs of producing and delivering them, and so on. Similarly, assessing the all-things-considered benefits of a carbon tax would require expertise in economics *and* public policy *and* climate science. None of us have such expertise across the range of policies we support. Such knowledge isn’t required for support of a policy to be epistemically justified.

I take it, though, that your beliefs about these policies are (usually) justified. In some cases—mainly, though perhaps not exclusively, when they concern uncontroversial policies—these beliefs might amount to knowledge. These beliefs are justified on the basis of *testimony*. The testimony that justifies your beliefs in these cases might be the testimony of people who you’re justified in believing *do* understand the causal mechanisms. Perhaps more commonly, it’s the testimony of those who you’re justified in believing are plugged into networks of informants, where this network contains many different people who together understand the relevant causal mechanisms. When it’s a political policy, you adopt it on the grounds that parties have mechanisms for integrating expert judgment, from a variety of different fields, in a manner that is guided by and reflects the values the party represents.

In all these cases, your understanding of the relevant mechanisms is typically irrelevant, and the discovery that your grasp of them is shakier than you’d thought should not be a defeater. Rather, a shaky grasp of the mechanisms involved is what you should expect: this stuff’s *hard* and we’re not experts on it. Expert judgment is routinely far more reliable than lay judgment, such that when we discover that our judgment conflicts with that of the experts, we usually have strong grounds for substituting their judgment for our own.^7^ Even when our support for some policy rests in part on our own understanding of the mechanisms involved, the realization that our understanding is weak shouldn’t measurably reduce our confidence. Given the relative unreliability of lay understanding in any case, our justification never really depended on it. The realization that we don’t understand the mechanisms should lead us to rethink the *grounds* for our belief, rather than lowering our *confidence* in it.

Of course, expert judgment isn’t always reliable. Some domains are expertise conducive; others are not. Expertise conducive domains are those in which inaccurate predictions are clearly and rapidly falsified, such that feedback is reliable, and causal mechanisms can be isolated sufficiently well. These conditions are satisfied to a greater or lesser degree across many domains, but far from all (Tetlock & Gardner, 2016). It’s not implausible that some of the policies that the participants in these experiments were asked to consider come from domains in which expert judgment is not very much better than lay judgment. But there’s no reason to think that participants are sensitive to this fact. If they were, we’d expect to see less partisan polarization on questions on which expert judgment is very much more reliable than lay judgment, like climate change and the efficacy of vaccines, than on those on which expert judgment is less reliable. Of course, we see no such thing; the reduction in confidence occurs both in domains in which expert testimony grounds lay knowledge and where it is too unreliable to transmit knowledge. In the second case, a reduction in confidence might genuinely improve our epistemic condition, since we formed our belief on the basis of testimony that wasn’t reliable. But the reduction in confidence also occurs in too many domains in which we lose (often valuable) knowledge.

Might the induction of IH actually play a role in bringing people to reject expert testimony outside the lab? It’s hard to be sure, but it’s likely because there are plenty of opportunities: political debate and media coverage of complex topics might often lead people to recognize they don’t understand the issues as well as they’d thought. Equally, debates between proponents of rival views might lead the audience to realize the details are beyond the non-specialist. If we think that we justifiably adopt beliefs in line with expert testimony only when we also understand the evidence for ourselves, exposure to debate might lead to agnosticism in the face of expert consensus (Why do we nevertheless often see high confidence in beliefs like climate change scepticism? Perhaps the induction of IH has different effects on scientific and moral or political testimony. Such a hypothesis isn’t implausible, since we might reasonably see moral or political expertise as depending on a kind of insight, rather than on a grasp of causal mechanisms. Perhaps, then, the realization that we don’t grasp the causal mechanisms leads to agnosticism on whether scientific testimony is true, but is consistent with high confidence grounded on testimony from *political* elites).^8^

The discovery that bringing people to appreciate the shakiness of their grasp of causal mechanisms leads them to weaken their support of policies urged by experts seems to confront us with a troubling possibility: To preserve support for policies that we regard as necessary, we might be required systematically to suppress public awareness of the details of the mechanisms; that, in turn, would require draconian restrictions on political debate. That would surely be a bad upshot. Fortunately, there’s an alternative: we should recognize that the induction of humility is epistemically pernicious only given certain – false – background beliefs, and it’s these beliefs we should aim to alter.

## Excessive humility?

I’ve argued that the humility induction used in these experiments leads people wrongly to lower their confidence in their beliefs, and that they’re wrong to do so because these beliefs are justified on the basis of testimony, and not (to any significant extent) on their own understanding of the causal mechanisms at issue. That’s bad. It’s also surprising: it’s precisely the opposite of what we would expect to result from IH. IH is supposed to be what Battaly (2018) calls an *effects-virtue*; that is, a virtue that has good epistemic effects (it might *also* be what she calls a responsibilist or a personalist virtue). Among its salutary effects is supposed to be the way in which it leads those who possess it to defer to others. The humble person recognizes her limitations and the epistemic superiority of others in domains in which *they* have expertise, and *she* does not; accordingly, she defers to them in those domains. If IH should lead to apt deference, it shouldn’t also cause agents to decrease their confidence in their judgments in cases like these, because these cases are squarely in domains (taken to be) the province of experts. This thought suggests that the problem isn’t IH at all. It’s only because we – arrogantly – take ourselves to be able to assess these complex issues for ourselves that the induction reduces our confidence.

On this interpretation of these cases, the root cause might after all be insufficient IH. While participants in the experiments were *appropriately* humble in accepting that their own understanding wasn’t sufficient to ground confident belief, they were also arrogant in clinging to their assumption that it’s *their* understanding that’s relevant. Different accounts of IH could cash this thought out in different ways: as arising from insufficient IH, or sufficient IH combined with an epistemic vice. Perhaps IH hasn’t permeated through agents’ cognitive economy. On the account of IH defended by Whitcomb et al. (2017), the experiments might illustrate how appropriate IH can coexist with arrogance. Perhaps the participants own their limitations, just as an intellectually humble agent should (according to Whitcomb et al.), but are arrogant about their strengths.

If this sort of interpretation of how the IH induction works is correct, my claim that there’s too much IH around would be false. On one kind of account, the problem would arise from an insufficiency of IH. On another, the problem isn’t IH at all, but the vices it coexists with in some individuals. Perhaps, as we might have suspected pretheoretically, the problem is epistemic vice after all.

But there’s a more parsimonious explanation. It’s more parsimonious because its rivals depend on a further assumption, and that further assumption can explain the phenomenon without invoking epistemic vice. Neither insufficient, but domain specific, IH nor IH coupled with arrogance explain the loss of knowledge as a consequence of the induction of IH unless we also attribute a false belief to the participants, and that belief is sufficient to explain the loss of confidence seen. It's only if we attribute to participants the mistaken belief that responsible agents accept only propositions that they have carefully assessed for *themselves* that we can explain the loss of confidence they manifest (I will call this commitment ‘epistemic individualism’).^9^ They must take their attitude to rest on their own assessment of the evidence (now shown to be inadequate) or at any rate be committed to thinking that it *should* rest on their own assessment. But this belief would give rise to the loss of confidence even if IH was consistent and unopposed in the person.

Of course, there’s nothing surprising about the possession of such a mistaken belief: the view that epistemic responsibility in this domain requires careful scrutiny of policies for oneself is widely held and reinforced in the broader culture, and (for that matter) in philosophy. Most thoughtful people probably don’t think that laypeople should ‘do their own research’, in the sense of studying economics or climate science before making their minds up, but they do think that epistemic responsibility requires some sort of grasp of the issues; perhaps from reading the sorts of summaries and sources accessible to laypeople that set out why these policies are recommended (for a single example, see Cassam’s (2018) argument that the responsible agent refutes a Holocaust denier by reading Wikipedia and the like; see Ballantyne et al., 2022; Levy 2022 for discussion). It’s because the induction shows that the person lacks this level of understanding that the loss of confidence occurs.

No doubt the layperson who has carefully read a broad selection of such sources and retained the information would know more than most of us about these policies. But they wouldn’t in fact be justified in their attitudes to them on the basis of their own grasp of the mechanisms. The sources accessible to laypeople can’t convey the grounds for acceptance of a policy in the sort of way that genuinely justifies it (Vickers, 2023; Levy 2022). As Vickers says, whilst this kind of information “might be nice to have—it gives me a warm, fuzzy feeling inside—that evidence should not play a role in persuading me, since it remains so impoverished compared with the full evidence base as documented in the scientific journals” (90).^10^ A good grasp of the accessible information is surely laudable, and increases our understanding, but is irrelevant to justification. Still, since most people (including experts) cling to the view that epistemic responsibility requires grappling with this kind of evidence, participants should be expected to share this belief. That explains the effects observed: The induction of IH lowers their confidence not because IH doesn’t permeate throughout their cognitive economy or because they’re arrogant about their strengths, but because they’re appropriately humble *given* their beliefs about how responsible agents behave. I take these thoughts to show that the problem isn’t inconsistent IH, or IH combined with an epistemic vice.

A more plausible thought is that the best explanation of the results doesn’t indict IH at all; rather, it’s the mistaken commitment to epistemic individualism that’s the root problem. *Given* that commitment, the induction of IH had precisely the effects it ought to have. That is, a reduction in confidence is called for, if we accept that we ought to base our beliefs concerning such topics on our own assessment of the evidence. The problem isn’t an excess of IH at all, on this view, but epistemic individualism. This thought can be developed in two different ways, one more plausible than the other. One version holds that the induction actually improved agents’ epistemic position. On this view, while it might be true that agents shouldn’t base their attitudes on their own assessment of the evidence, they actually do so, and induction of IH therefore improved their epistemic position by bringing them to see that that assessment was far from adequate. On this view, IH functioned as an effects virtue, just as it should. In lowering their confidence, participants improved their epistemic position: they recognized that their actual grounds couldn’t do the work they had supposed. So there’s no excess of IH; there’s just the right amount.

But that’s not right: IH doesn’t improve participants’ epistemic position in the experiments. It worsens it. It worsens it, because in fact participants’ attitudes toward policies is *not* based on their own assessment of the causal mechanisms involved. It’s based on testimony, just as it should be.

There are two reasons to think that participants’ beliefs are in fact based on testimony. First, there’s the partisan clustering of these beliefs. If you know a person’s ideological leanings, you can predict with relatively high confidence what their attitudes to very many policies will be (Joshi, 2020). We shouldn’t expect to see this kind of clustering if attitudes were based on people’s own assessment of the causal mechanisms involved. We should instead see much more variability in attitudes. The best explanation of why policy attitudes cluster is that these attitudes are based (directly or indirectly) on the positions signalled by the political elites with whom people identify (e.g., Holcombe, 2021); and such cues constitute (and reflect) testimony.^11^

The second reason to doubt that people really do base their attitudes to their policies on their assessment of the causal mechanisms involved is empirical. Ironically, this empirical data stems in part from the work of some of very same researchers who see the humility induction as salutary. This evidence demonstrates the extent to which people implicitly defer to others, as well as a dissociation between this implicit deference and their explicit theories.

Knowledge and belief are largely social achievements: apart from what we know about our immediate environment and the people with whom we interact everyday (and these beliefs are only a partial exception), we know almost everything we know on the basis of testimony. I’ve never been to Bogatá, or Budapest or Bangkok, but I know these cities exist; I’m forever cut off from direct contact with the siege of Leningrad or the sack of Rome, but I know these events took place. I’ve never seen a smallpox virus, or a black hole; once again, I know these things are real, and I’m confident in the germ theory of disease and the Big Bang theory of the origin of the universe. I know all these things on the basis of testimony, testimony from informants I have very good reason to regard as reliable. Everyone else is in the same boat: even scientists know much of what they know *about their own speciality* very largely on the basis of testimony. A developed science is far too complex for the individual scientist to be able to gather all the evidence, let alone develop all the theories she needs, for herself. Instead, the individual scientist, too, plays her part within a distributed network of informants, while trusting that others play their part too (Elgin, 2011).

People are *implicitly* aware of how deeply social knowledge is, but they don’t explicitly recognize the depth of their epistemic dependence on others. Rabb et al. (2019) review evidence that people’s assessment of their *own* ability to answer factual questions without using the internet is increased when the internet is accessible to them, compared to when it is not. The availability of a source of reliable testimony, that is, inflates people’s perception that they do not need to call upon it. Similarly, people rate their own capacity to explain natural phenomena as higher when they are told experts understand the phenomena than when they are told they do not (Sloman & Rabb, 2016), and they rate their own understanding of psychiatric disorders higher when they believe that society understands them (Zeveney & Marsh, 2016). We take ourselves to know on our own when our community, or experts within it, know. In fact, the confusion between what the community knows and what *we* know on our own is likely responsible for the original illusion of explanatory depth: we take ourselves to understand something when we don’t because we are implicitly aware that experts, or the community at large, understands it (Keil & Wilson, 2000).

In the light of this evidence, we should be confident that people do in fact rely very heavily on testimony. Taken together with the ideological clustering of attitudes, we should think that the IH induction *inappropriately* lowers their confidence: they were justified in their attitudes prior to it. It is for this reason that I think it’s plausible to maintain that we may lose knowledge due to an excess of IH. It remains true, however, that it is epistemic individualism that is the fundamental culprit. If we accepted, in theory, and not just (albeit somewhat inconsistently) in practice, that beliefs about questions which fall significantly within the purview of expertise must be justified on the basis of testimony and not non-expert assessment of the evidence, then the induction of IH wouldn’t cause a loss of knowledge. For agents with more accurate beliefs concerning when we ought to defer, IH might be an effects virtue after all.

A final objection might be empirically based. In recent work, Meyer and Alfano (2022) provide evidence that those who endorse flagrantly false conspiracy theories and those who are more likely to accept fake news also score lower on what they call epistemic humility. They measure epistemic humility using a scale that Alfano and colleagues had previously developed and validated (Alfano et al., 2017). If they’re right, the induction of IH might be a useful tool. Perhaps it’s a tool that should be used in a targeted manner, aimed at those who are particularly low in IH, so as not to risk knowledge or justified belief in those who are deferring appropriately and selectively, whatever their explicit beliefs about responsible epistemic agency.

This suggestion is, it should be noted, compatible with my claim there’s too much IH about. There might be too much IH about and also true that some subgroup suffers from too little IH.^12^ Our conclusion wouldn’t be quite as revisionary, in that case, since we’d continue to think that IH is an epistemic virtue we ought to inculcate or induce in others. Nevertheless, before we accept this qualification to my claims, we should examine Myer and Alfano’s argument carefully.

In a response to Myer and Alfano, Klein (2022) notes a puzzle: conspiracy theorists present themselves as open-minded and engaged with evidence, rather than epistemically arrogant. He also notes that there is some evidence that they really *do* engage with evidence (Klein et al., 2018; Lee et al., 2021). Klein suggests reconciling the (admittedly indirect and weak) evidence that conspiracy theories really do engage with evidence with Myer and Alfano’s findings via the hypothesis that they’re *performing* the virtues, rather than actually possessing them. I want to suggest a quite different explanation, according to which conspiracy theorists are no less epistemically virtuous than anyone else.

There’s a major problem confronting research into conspiracy theories and the like: it’s hard to be confident that everyone is reporting their genuine beliefs. Scepticism is cheap, of course, but here it’s motivated: there’s good evidence that people often don’t report their true beliefs on these kinds of questions. Just the content of some of these reports should give us pause: do we really find it plausible that one in four Americans genuinely believe that Barack Obama might be the Antichrist (Harris, 2013)? There is also extensive experimental evidence for a gap between people’s reported beliefs on partisan issues and their actual beliefs (Hannon, 2021). For instance, perceptions of the state of the economy tracks the party of the President: when the President belongs to the rival party, political partisans report the economy is doing worse than when their party is in charge—but their economic behavior doesn’t seem to reflect their reported perception (Bullock & Lenz, 2019). Further, incentives to report accurately reduce the partisan gap in responses (Prior et al., 2015). Most compellingly Schaffner and Luks (2018) found that Republicans were significantly more likely than Democrats to report that an (unlabelled) photograph of the Trump inauguration depicted a larger crowd than a similar photograph of the Obama inauguration (see Ross & Levy, 2022 for a replication). It’s really not plausible that they truly believe that the first photo depicts a bigger crowd; rather, they’re expressing their support for one side of politics.

In addition to reports aimed at expressing support for one side of politics (or signalling belonging; Ganapini, 2021), people sometimes engage in sheer trolling (Lopez & Hillygus, 2018). They may report beliefs they find entertaining or outrageous or ‘edgy’. The blogger Scott Alexander has proposed a ‘lizardman constant’; a proportion of individuals who will consistently but insincerely answer ‘yes’ to the question “are lizardmen running the earth?” (Alexander, 2013). I doubt there’s any such constant: the extent of trolling will depend on properties of the population probed and the questions asked (for example, Republicans may troll psychologists because they perceive that a survey is designed to make their side of politics look irrational or ill-informed).

It’s very likely that some of the respondents Meyer and Alfano probed engaged in insincere response. Most of the items they utilized were plainly coded politically, such that they offered respondents an opportunity to express their support for one side of politics or another. One of the 5 conspiracy theories they put to respondents was the notorious ‘Birther’ conspiracy, according to which Obama was not born in the United States; almost certainly some people report endorsing that view not because they believe it but to express opposition to Obama. Similarly, acceptance of fake news was assessed using items like this headline from *Infowars:* “Revealed: UN plan to flood America with 600 million migrants;” again, it’s likely some respondents falsely report believing it to express support for their side of politics. This sort of worry applies to all but (at most) one fake news item: each presented respondents with an opportunity to signal support for their side of politics. Every conspiracy theory presented was bizarre or outrageous enough to elicit some degree of trolling from some respondents, regardless of their political commitments.^13^ There’s a dilemma that confronts all research on this kind of topic: make headlines too plausible or too mundane, and the case for thinking that acceptance of them manifests vice weakens considerably, but make them too implausible or bizarre, and the case for thinking that respondents are sincere weakens instead.

No doubt many, probably most, of the respondents responded to most of the items sincerely. But we need grounds for confidence that the sincere responders drive the findings. Describing their second, preregistered, study, Meyer and Alfano claim that “[a]fter controlling for other variables, intellectual vice accounts for 10–13% of the variance in conspiracism and acceptance of fake news” (252). That’s a decent chunk of variance, but further work is needed to show that this result is due to a difference between sincere responders and not due to insincere responding. Analogous problems arise with regard to the epistemic humility scale. The scale items are fairly transparent: most probe dispositions that are widely held to be (dis)valuable (“I enjoy reading about the ideas of different cultures”; “I don’t take people seriously if they’re very different from me”). At least some proportion of people will report that they possess these dispositions because they know that they’re held to be valuable, and especially held to be valuable by those with whom the respondents identify. Some will probably report that they lack such dispositions for the purposes of trolling, or because they take these to be dispositions valued by those on the left.

Admittedly, as part of their validation for their scale Alfano et al. (2017) compared self-reports to reports by informants who know the person well, and found reasonable correlations. But the correlations were lowest for open-mindedness and engagement, the virtues most associated with endorsement of conspiracy theories and fake news, and the number of informant reports they were able to gather—107—was small. It is also possible that some middle position is true: that the vices they measure predict not *belief* in conspiracy theories or fake news, but *willingness to endorse* such theories, perhaps for the purposes of trolling or for expressive responding. It’s important to note that belief in conspiracy theories and the like is easily explained in ways consistent with IH. Those who accept them might do so on the same kind of basis as those who reject them: on the basis of testimony from those they see as trustworthy and competent.

It might be that those who accept blatantly false beliefs manifest a deficiency in IH. Perhaps they’re arrogant, and substitute their own judgments for those of authorities they should trust. If that turns out to be the case, then *they* act as we believe *we* ought to: making up our own minds and decreasing confidence in our beliefs when we discover our own understanding of the issues is insufficient to justify them. Would that be grounds for condemnation of their lack of IH? It’s not obvious that it’s arrogant to govern oneself as you judge you ought.

## Responding to the problem

I’ve argued that agents lose confidence in attitudes that are actually justified for them when they recognize that their own understanding is inadequate to ground these attitudes. I’ve claimed that in doing so they manifest IH, and that therefore IH is apt to cause a loss of justified belief under certain conditions. This is my evidence for the claim that there’s too much IH around: ordinary agents are disposed to lose confidence in justified beliefs because they possess the virtue. I’ve also suggested (more tentatively) that our excess IH may have played and be playing a significant role in promoting and stabilising unjustified beliefs. Once beliefs become entrenched as components of our identities, they’re resistant to such manipulations, but unjustified beliefs may become entrenched in the first place in part because those tempted by better beliefs were brought to see that their individual knowledge couldn’t justify them.

I’ve also conceded, however, that the root of the problem isn’t IH, but our theoretical commitment to epistemic individualism. Given that’s the case, the widespread view that we might address our epistemic predicament (inter alia, of course) by seeking to inculcate IH seems to miss the mark. We do far better to address our *theories*. We need to reject our pervasive epistemic individualism.

We’re epistemically social animals: in every sphere of life, we’re dependent on others for navigating the world and achieving our goals. We smoothly and flexibly integrate testimony and all kinds of implicit information, provided by others and the environment we’ve shaped together, into our cognition: we *live* our epistemic interdependence. But we couple the disposition to think socially with an epistemic individualism: we believe that we ought to accept only what we have adequately scrutinized. Most of the time, this belief is inert. Perhaps it belongs to the class of belief that Sperber (1997) calls *reflective*; beliefs that we accept on reflection but which don’t govern our intuitive cognition. But even if their scope is limited, reflective beliefs are not epiphenomenal: reflective beliefs govern cognition when they’re activated and—especially—when activation is combined with attention to the unfolding of thought.

Reflective beliefs certainly have an influence on our cognition and therefore our behavior: when we’re reminded to reflect—by other agents, or by finding ourselves in the kinds of circumstances in which such cognition is expected of us (e.g., when we’re confronted by legalese)—it exercises cognitive sway. Consider choosing a candidate to vote for. This kind of non-habitual, high-stakes, decision certainly triggers reflection for many. The induction of IH is likely a cue that switches most of us into reflective mode and thereby makes our epistemic theories causally efficacious. The induction breaks the flow of intuitive thought. When we’re immersed in cognition, we *live* our epistemic interdependence, but when the flow is broken, we step back and examine it (if you like, it transforms thinking from the ready to hand to the present at hand; Heidegger, 2010). When we’re engaged in this mode, our theories about thought are active, and our recognition that our attitudes toward policies are unjustifiable on the basis of these theories reduces our confidence in these attitudes, with downstream effects on further thought and behavior. Bad actors can weaponize this switch, inducing reflective thinking in order to bring us to reject beliefs that are actually justified for us.^14^

The most straightforward, and perhaps the only practical, way to address our loss of justified belief through the induction—and weaponization—of IH is to address our epistemic individualism. As we saw, the prospects for recalibrating the virtue are poor, and we certainly don’t wish to avoid reflection. Reflection is obviously central to much of the cognition we rightly value most. We need instead more fully to recognize our epistemic interdependence, so that we don’t merely live it, but we see it as reflectively justified. How do we reach ordinary people with this message? It may not be as difficult as it seems. To some extent, how difficult it is might depend on its origins.

Why do we accept a theory at odds with our epistemic practice? There are two possibilities. One is that this individualism is actually epistemically adaptive: thinking we’re able to figure things out for ourselves motivates us to engage in epistemic exploration that is hubristic, but nevertheless enables us to contribute to the shared epistemic project far more effectively than if we had accepted our epistemic limitations. If that’s right, epistemic individualism might be innate, or at least developmentally canalized; that is, tending to develop reliably under a wide range of environmental conditions, including those to which developmental trajectory is in other respects sensitive (see Mercier & Sperber, 2011, 2017; Levy 2019 for suggestions along these lines). The other possibility is that our epistemic individualism is a product of culture. Perhaps it’s a recently emerging product of the Enlightenment. Perhaps it has deeper historical roots, in the same set of forces that produced the individualism apparently characteristic of WEIRD (western, educated, industrialised, rich and democratic) people (Henrich, 2020). These two developmental stories have different implications for how easy it might be to uproot epistemic individualism, and about the costs of doing so.^15^

If the first story is correct, it is likely to prove more difficult to shift us away from epistemic individualism, since developmental canalization predicts resistance to environmental influence. Moreover, attempts to do so might have epistemic costs. It’s worth noting, however, that the correlation between developmental canalization, on the one hand, and difficulty and costs, on the other, is only rough. Developmental canalization entails resistance to a broad range of environmental perturbations, but it might be possible to find one that works; it need not always be one that is difficult to implement, and it might turn out that the epistemic individualism that was adaptive earlier in cultural or genetic evolution is no longer adaptive in our current environment. Still, if this story is correct, we may face obstacles we should bear in mind.

If our individualism is more deeply cultural, it might be easier to change, and the epistemic costs (if any) might be lower. Again, there are no entailments here: a disposition (or other phenotypic characteristic) might be due to environmental factors like culture, yet very hard to shift, or very hard to shift without incurring unacceptably high costs. If our epistemic individualism is distinctively WEIRD, however, then we have other cultures as models of how it might be reduced without (apparently) risking things we rightly value. Prima facie, less individualistic cultures can promote flourishing as well as WEIRD cultures. It may be that philosophy has a role in countering epistemic individualism that is greater than it usually has in addressing important ills. While it is unlikely that philosophy has been solely or even mainly responsible for epistemic individualism, it may have played a role in its development and its spread. Philosophy may also have a role to play in rolling it back; in bringing us to see that our *actual* deference is *theoretically* justified.

## Conclusion

Friends of IH rightly value deference: when others are better placed to answer some question than we are, we have a strong (though of course defeasible) reason to defer to them. It would be arrogant to substitute our own judgment for that of those more expert than us. It’s seemingly paradoxical, then, that inducing IH at least sometimes causes people to *reject* expert testimony. I’ve suggested that the induction of IH works by bringing us to engage in effortful, conscious, deliberation, and that when we engage in such deliberation our reflective beliefs (which otherwise tend to be inert) play a pivotal role in cognition. Since we hold an explicit theory according to which we should accept important propositions only if we have assessed the issues for ourselves, when explicit cognition drives thought we reduce our confidence on difficult topics, with further consequences for our thought and behavior.

Philosophy, with its emphasis on thinking for oneself, may have played a role in making epistemic individualism so widely accepted. It may also play a role in promoting a culture in which IH is induced: in which people are challenged to think for themselves before accepting testimony. But philosophy can also be part of the solution: promoting better theories. Of course, thinking for oneself is important, but when we lack the expertise to assess complex topics for ourselves, thinking for oneself should be in the service of better deference, not an alternative to it. IH is not the solution to our epistemic predicament; we seem to have more than enough of it as things are. Better theory, not virtue, is the way forward.^16^

## Data Availability

NA.

## Footnotes

1 There is some debate in the literature whether arrogance and humility are indeed contraries. On one influential view (Whitcomb et al., 2017), they can and do coexist in individuals. I don’t need to take a stand on this issue: the thought that our epistemic predicament is caused by a lack of IH is a claim about what vices or dispositions actually underlie epistemic ills; it doesn’t entail or require that arrogance can’t coexist with humility. For reasons of stylistic convenience in what follows I will sometimes use “arrogance” to refer to a lack of IH.

2 See Snow (2019) for a close examination of the major accounts of IH.

3 Strictly speaking, this is Driver* and not Driver. Driver herself was writing about modesty, not humility, and she takes modesty to be subtly different to humility. Moreover, she wasn’t concerned specifically with an epistemic or intellectual quality. Nevertheless, her theory has been elaborated into a view of epistemic humility by a number of writers (e.g., Priest, 2017; Tanesini, 2018; Whitcomb et al., 2017), none of whom find it plausible.

4 In her most recent discussion of IH, Tanesini (2021b) argues that it is underpinned by attitudes, rather than dispositions. I assume, however, that she would accept that whatever the fundamental ontology of intellectual virtues and vices, they entail dispositions to behave in characteristic ways.

5 At least, all sides acknowledge that IH entails a willingness and a disposition to moderate confidence in an attitude in the light of *first-order* evidence. On Hazlett’s level-splitting view, IH requires that we moderate our higher-order attitudes to our beliefs in the light of higher-order evidence stemming from peer disagreement; it doesn’t require that we moderate our first-order attitudes in the light of higher-order evidence (Hazlett, 2012).

6 After their paper was written, Joe Biden announced that student debt would be reduced. That announcement has dragged the issue to the centre of partisan debate, and nothing is more likely to bring it about that a policy moves from the consequentialist category to the identity category.

7 Of course, when a policy is controversial this may be because experts come to conflicting conclusions about it. Perhaps expert disagreement should be a defeater for your support for a policy. But your grasp of the mechanisms involved is once again irrelevant (indeed, if the experts disagree, you should be even more confident that you’re not in a position to evaluate the mechanisms for yourself). I believe that we may be justified in deferring to experts on one or other side of a controversy on value-laden grounds, even in the absence of some other way to choose between them (Levy, 2021), but you needn’t accept that claim to accept that whether or not expert disagreement is a defeater, our own understanding of the relevant mechanisms is irrelevant in these kinds of cases.

8 An alternative hypothesis is that the realization that we can’t justifiably assess an issue for ourselves leads to a kind of epistemic nihilism, according to which any belief is as good as another, and we might as well adopt the beliefs that align with our partisan affiliation.

9 Strictly speaking, it is probably more accurate to see acceptance of the belief that the possession of knowledge requires some kind of scrutiny of the first-order evidence for oneself as a *manifestation* of epistemic individualism rather than as constituting it; epistemic individualism might be regarded as a pervasive and systematic undervaluing of social and collective sources of knowledge and epistemic agency.

10 Vickers is discussing scientific theories here, but the point obviously generalizes to policy questions, since they routinely depend on such theories.

11 Of course, many people don’t have strong ideological leanings. But those people without strong ideological leanings are also much less likely to have confident attitudes toward polices. It is worth noting that experts, for whom deference plays a smaller role in attitude formation, also exhibit ideological clustering of attitudes. An important part of the explanation for their clustering turns on the ways in which the understanding of mechanisms is itself normatively inflected. But nothing like this holds true for most of us. Rather than our grasp of causal mechanisms being inflected by our values, we have little grasp on the causal mechanisms in the first place.

12 Tanesini (2021b) emphasises the social determinants of virtues and vices; servility, for example, has as one of its causes being held in low regard by others. On this basis, we might expect excessive IH not to be general but confined to marginalized populations. That might be the case, but it seems unlikely that the experimental evidence is driven by such individuals. Those who are lower than the mean in IH are also less likely to claim confidence in their attitudes in the first place. Moreover, the illusion of explanatory depth is robust across different populations with different demographic profiles (Rozenblit and Keil, 2002).

13 I’m grateful to Mark Alfano and Marco Meyer for sharing their prompts with me.

14 I’m not suggesting, of course, that merchants of doubt need conceptualize their activity in this kind of way. When they seek to justify it to themselves, they (too) are in reflective mode, and may see the beliefs they undermine as actually unjustified.

15 It’s likely that we’re epistemic individualists for the same reasons we’re individualists in other domains; the rich feminist literature on relational autonomy and relational concepts of the self might provide us with clues to the origins of our individualism and also guide us in addressing its common causes across multiple domains. For feminist models of relational autonomy see the essays collected in Mackenzie and Stoljar (2000), and the editors’ introduction to the volume; Elzinga (2019) has developed a relational model specifically designed to account for epistemic autonomy. Code's (1987) relational model of personhood has been widely influential.

16 I am very grateful to two reviewers for this journal for a challenging and helpful set of comments, working through which genuinely improved the paper.
